# Factors affecting low coverage of the vitamin A supplementation program among young children admitted in an urban diarrheal treatment facility in Bangladesh

**DOI:** 10.1080/16549716.2019.1588513

**Published:** 2019-05-07

**Authors:** Ishita Mostafa, Shamin Fatema Islam, Prasenjit Mondal, A.S.G. Faruque, Tahmeed Ahmed, Md Iqbal Hossain

**Affiliations:** a Nutrition and Clinical Services Division, International Centre for Diarrhoeal Disease Research, Bangladesh, Dhaka, Bangladesh; b Faculty, James P. Grant School of Public Health, BRAC University, Dhaka, Bangladesh

**Keywords:** VAD, vitamin A deficiency, VAS, vitamin A supplementation, 1–5 -year -old children, parents without formal schooling, wealth quintile

## Abstract

**Background**: Vitamin A deficiency (VAD) is one of the most prevalent micronutrient deficiencies in the world. About 2% of all deaths among children under five years of age (U-5) are attributable to VAD. Currently evidence-based knowledge is grossly lacking about the factors associated with low coverage of VAS.

**Objective**: This study aims to determine the factors affecting low coverage of the vitamin A supplementation program among the young children admitted to a diarrheal hospital.

**Methods**: We extracted data from the Diarrhoeal Diseases Surveillance System (DDSS) on children aged 12–59 months admitted to the Dhaka Hospital of the International Centre for Diarrhoeal Disease Research, Bangladesh, from 1996 to 2014. A logistic regression model was constructed to identify the factors that were significantly associated with non-compliance to vitamin A supplementation (VAS). Strength of association was determined by calculating adjusted odds ratios (aORs) and their 95% confidence intervals.

**Results**: A total of 8649 children were enrolled and comprised the analyzable sample. Their mean ± SD age was 25.2 ± 12.8 months and 40% were female. Around 68% of them had received VAS in the previous 6 months. In the logistic regression analysis, older (>24 months) children (aOR: 1.38; 95% CI: 1.24–1.53), having an illiterate mother (aOR: 1.43; 95% CI: 1.27–1.64), having an illiterate father (aOR: 1.3; 95% CI: 1.16–1.50), coming from the two lowest wealth quintiles (aOR:1.13; 95% CI: 1.02–1.27), with an average monthly household income <10,000 BDT, (1 USD = 60 BDT) and children who had not received the measles vaccine (aOR: 1.87; 95% CI: 1.63–2.19) were more likely not to have received VAS in the preceding six months. We also observed an increase in coverage of VAS from 61% to 76% over the last 18 years (p < 0.001).

**Conclusions**: Non-compliance to VAS was found to be associated with older children, parents without formal schooling, family with greater poverty, low family income, and lack of measles vaccination. Specific programmatic approaches including prioritizing vulnerable children may enhance vitamin A coverage.

## Background

Vitamin A is a crucial micronutrient for proper functioning of the visual system, growth and development, maintaining the integrity of epithelial cells, immune competence and reproductive ability [1,2]. Vitamin A deficiency (VAD) is one of the most prevalent micronutrient deficiencies in the world. Infants and pregnant women living in low- and middle-income countries are most at risk of developing the deficiency. About 2% of all deaths of children under five years of age (U-5) are attributable to VAD [3]. Symptoms of deficiency include anemia, impairment of immune function leading to increased risk of morbidities in children, and night blindness [1]. Currently, VAD is considered a major cause of preventable childhood blindness [4].

Globally 5.2 million children are suffering from night blindness, one of the earliest noticeable clinical features of VAD, which is usually irreversible. Additionally, 9.8 million pregnant women, who constitute 8% of the population at risk of VAD, are affected by the same condition. In Bangladesh, prevalence of night blindness among U-5 children decreased from 3.5% in 1982–83 to 0.5% in 2004 [5]. Serum retinol concentration is another measure for detecting VAD in the population, and a value of <0.7 mmol/L indicates sub-clinical VAD, even in the absence of apparent clinical symptoms. Low serum retinol is prevalent among 190 million U-5 children, as well as among 19 million pregnant women [6]. In Bangladesh, a national survey reported that 40% of non-pregnant non-lactating (NPNL) women, 20.5% of preschool aged children (PSAC) and 20.9% of school aged children (SAC) suffer from this condition. The proportions are even higher (38% in PSAC and 27% in SAC) in the slum areas [7].

The most important cause of this deficiency is inadequate intake of vitamin A-rich foods. In Bangladesh, U-5 children consume 270 retinol equivalents (RE) of vitamin A per day, against the recommended daily allowance (RDA) of 300 RE. Alarmingly, animal-source retinol, which is highly bio-available, constitutes only 20% of that intake. The scenario is worse for women of child-bearing age. Their daily intake is 372 RE compared to their RDA of 700 RE, where animal sourcescontribute only 8% of their vitamin A intake [7].

Periodic supplementation with high doses of preformed vitamin A among vulnerable age groups (PSAC, SAC and NPNL women) is a commonly adopted strategy in low- and middle-income countries like Bangladesh to alleviate the vitamin A status of general population. The supplementation program for children is often coupled with national health campaigns such as immunization. Since VAS with a higher dose can retain improved vitamin A status for only up to 3 months, such a program must primarily focus on improving immune function and reducing mortality associated with measles, diarrhea, and other illnesses [4]. Two systematic reviews suggested that regular VAS resulted in a 24–28% reduction in mortality as well as a decrease in the incidence of diarrhea and measles and the prevalence of vision problems, including night blindness and xerophthalmia [8,9].

However, findings on vitamin A coverage from a 6-month VAS program are not consistent in Bangladesh. According to a micronutrient status survey conducted in 2011, it was seen that only 77% of the families with U-5 children reported receiving VAS in the preceding 6 months [7]. According to the Bangladesh Demographic and Health Survey (BDHS) conducted in 2014, only 62% of children had received VAS in the preceding 6 months [10]. To reduce the prevalence of vitamin A deficiency, the Directorate General of Health Services, Bangladesh, set a target to achieve vitamin A coverage of 90% by 2016 through its Health Population Nutrition Sector Development Program [11].

Currently, evidence-based knowledge is grossly lacking about the factors associated with low coverage of VAS. Thus, it has become imperative to address this knowledge gap as well as to suggest measures to enhance the coverage of VAS. This analysis aims to find out the reasons behind this non-compliance among young children aged 1–5 years who sought care in a large diarrheal disease facility. The results of this analysis can be of immense importance for policymakersto enable them to plan new strategies to bring the ‘non-compliant’ children under coverage.

## Methods

The Dhaka Hospital of the International Centre for Diarrhoeal Disease Research, Bangladesh (icddr,b), located in Dhaka, the capital city of Bangladesh, has provided care and treatment to people with diarrheal diseases since 1962. Data for this secondary analysis were extracted from the DDSS. The DDSS was established in 1979, in the Dhaka Hospital of icddr,b. DDSS is systematically sampling 2% of all patients, irrespective of age, sex, disease severity or socio-economic status. All related information was collected by research assistants by administering a structured field tested questionnaire. DDSS collects information on clinical, epidemiological and demographic characteristics, and the use of drugand fluid therapies at home. In addition, for children less than 3 years old, data on their feeding practices are collected. Information on their immunization and VAS status is also gathered. Children’s weight is measured to a precision of 0.1 kg, and height/length was taken with a precision of 0.1 cm by the research assistants. Z scores are calculated using the new child growth standards of the World Health Organization (WHO) [11]. Children with z scores < −2 standard deviations (SD) from the median for weight for height, weight for age and height for age are considered wasted, underweight and stunted, respectively. The socio‐economic status of the household was measured by scoring the household assets available from the data of the hospital surveillance system. DDSS monitors changes in patient characteristics as well as the changing pattern of diarrhea-causing enteric organisms [12]. For the present analysis, relevant information was extracted for children aged 12 to 59 months from the DDSS database of the Dhaka Hospital of icddr,b for the period 1996–2014.

## Statistical analysis

We used Statistical Package for Social Sciences (SPSS) for Windows version 20.0 (IBM Corporation, New York, NY, USA) for analyses of data. A wealth index was constructed based on ownership of selected assets, household structure (materials used for floor, roof and wall of the house), type of latrine used and source of drinking water. The wealth index was developed through principal component analysis and wealth quintiles were placed on a continuous scale of relative wealth from ‘poorest’ to ‘richest’ [13]. A Chi-square test was used to compare the differences in proportions of categorical variables. All independent variables were analyzed initially in a univariate model, and the attributes that were found to be significantly associated (p value <0.05) with the dependent variable (not receiving vitamin A supplementation in the last 6 months) and biologically plausible were included in the regression model. To establish logistic regression models we also checked for multicollinearity between independent variables using the variance inflation factor (VIF). In the final model, the VIF values of all independent variables were less than or around 2 and the mean VIF was 1.48. Strength of association was determined by calculating adjusted odds ratios (aORs) and their 95% confidence intervals (CIs). Independent variables included age and sex of the child, maternal and paternal level of education, immunization status, monthly family income, wealth quintile, and nutritional status of the child (weight for height, height for age, and weight for age). The Chi-square value was determined to check for a significant increasing trend of receiving VAS in the study children. A probability of < 0.05 was considered statistically significant.

## Results

**Figure 1.**
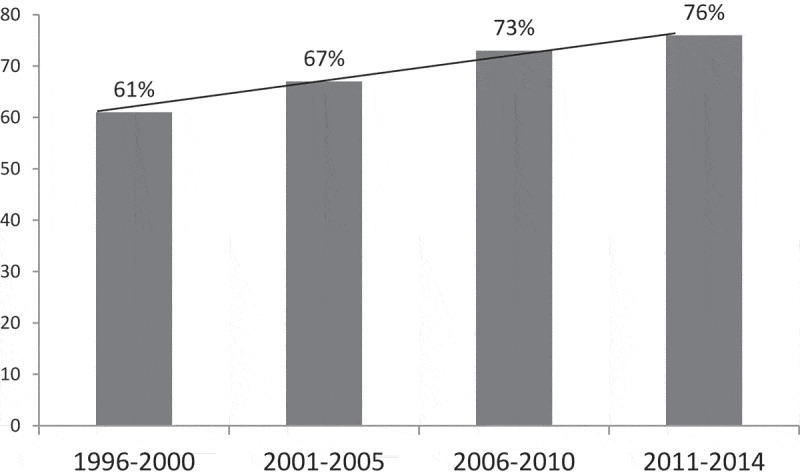
Percentage of children reported to the Dhaka Hospital of the International Centre for Diarrhoeal Disease Research, Bangladesh.

Data were extracted for 8649 children aged 12–59 months for the study period and they comprised the analyzable sample of the present study. Their mean ± SD age was 25.2 ± 12.8, months and 40% of them were girls. Around 68% had received VAS in the previous 6 months. The mean age of children who did not receive VAS was significantly higher than that of children who had received VAS in the last 6 months (28.9 ± 15.2 vs. 23.5 ± 11.0, p = 0.001). The proportion of parents without formal schooling was significantly higher among children who did not receive VAS than among those who received the supplementation (43% vs. 26% for fathers and 46% vs. 28% for mothers, p < 0.001). Significant differences were observed for socio-economic status of families and nutritional status of children with and without VAS (Table 1). Logisic regression analysis revealed older age, parental illiteracy, absence of measles vaccination, families of a lower wealth quintile and with low monthly income, and households of BDT less than 10,000 (1 USD = 60 BDT on average during the study period) were significantly associated with low vitamin A coverage in the last 6 months, after simultaneously controlling for covariates. Similar results were observed when different conditions of malnutrition (stunting, wasting, and underweight) were added in the logistic regression model (Table 2). A significant increasing trend in receiving VAS was also observed during the study period (61% to 76%; p < 0.001 over the last 18 years) (Figure 1).

**Table 1.** Factors affecting low coverage of the vitamin A supplementation program among children 12–59 months old admitted in the Dhaka Hospital of the International Centre for Diarrhoeal Disease Research, Bangladesh, from 1996 to 2014.

Variable	Did not receive vitamin A within last 6 months N = 2757 (%)	Received vitamin A within last 6 months N = 5892 (%)	P value
Children older than 24 months	1189 (43.1)	1748 (29.7)	<0.001
Female child	1134 (41.1)	2347 (39.8)	0.131
Wasting (WHZ < −2)*	953 (34.6)	1709 (29.0)	<0.001
Stunting (HAZ <\ −2)**	1309 (47.5)	2245 (38.1)	<0.001
Underweight (WAZ <−2)***	1453 (52.7)	2486 (42.2)	<0.001
Illiterate father	1175 (42.6)	1545 (26.2)	<0.001
Illiterate mother	1262 (45.8)	1622 (27.5)	<0.001
Did not receive Bacillus Calmette–Guérin (BCG) vaccine	152 (6.1)	92 (1.6)	<0.001
Did not receive diphtheria, pertussis, and tetanus (DPT) and polio vaccines	150 (6.0)	98 (1.7)	<0.001
Did not receive measles vaccine	446 (17.9)	497 (8.6)	<0.001
Monthly income less than 10,000 BDT (1 USD = 60 BDT, on average)	2196 (81.8)	4139 (71.2)	<0.001
Asset quintile			
Richest	470 (17.1)	1433 (24.4)	<0.001
Upper	516 (18.7)	1318 (22.4)	
Middle	449 (16.3)	1048 (17.8)	
Lower	651 (23.6)	1049 (17.8)	
Poorest	667 (24.2)	1037 (17.6)	

*Weight for height z ﻿score. **Height for age z score. ***Weight for age z score.

**Table 2.** Logistic regression showing associated factors to not receiving vitamin A supplementation among children 12–59 months old admitted to the Dhaka Hospital of the International Centre for Diarrhoeal Disease Research, Bangladesh, from 1996 to 2014.

Variable	OR (95% CI)
Age > 2 years	1.38 (1.24–1.53), p < 0.001
Female child	1.06 (0.96–1.17), p = 0.24
Illiterate mother	1.43 (1.27–1.64), p < 0.001
Illiterate father	1.3 (1.16–1.50), p < 0.001
Did not receive measles vaccine	1.87 (1.63–2.19), p < 0.001
Asset quintile (lowest two)	1.13 (1.02–1.27), p = 0.036
Monthly income less than 10,000 BDT (1 USD = 60 BDT, on average)	1.21 (1.09–1.41), p = 0.004
Weight-for height z score <−2	0.91 (0.86–1.07), p = 0.19
height-for age z score <−2	1.1 (0.93–1.197), p = 0.38
weight-for-age z score <−2	1.07 (0.92–1.24), p = 0.82

## Discussion

Due to a lack of any evidence-based knowledge, this secondary data analysis was carried out to determine the factors associated with non-compliance to VAS. This study revealed that non-adherence to VAS was mostly associated with parental education, and child’s age and measles vaccination status, as well as a poor socio-economic context of the household. Around two thirds of the study children had received VAS in the preceding 6 months, which is similar to the findings of the latest national micronutrient survey [7]. Our analysis showed that parental education is an important predictor of compliance with the VAS program, a finding that corroborates the results of a pooled data analysis in African settings [14]. A literature review has revealed any implementation of a door-to-door supplementation program resulting in universal visiting of households by the health work force may nullify the negative impact of paternal illiteracy [15]. Illiterate parents fail to understand the important health impacts of VAS, and thus they are less motivated to participate in the supplementation program. It can also be assumed that illiterate mothers lack appropriate information about vitamin A distribution campaigns, particularly time and place of distribution in the community, which might be an important reason for their not participating in supplementation programs, as reported by a previous study [15]. A study in a similar setting of India reported that children with illiterate mothers are at higher risk of not receiving VAS [16]. A study conducted in Cambodia found a significant association of low maternal education with lower VAS attendance in the preceding 6 months, although paternal education was not associated [17]. There is ample literature to suggest that a lack of maternal or caregiver education is an important barrier to VAS coverage [16,18].

Our study findings showed that older children were more likely not to receive VAS than their younger peers. Similar findings were reported in a pooled data analysis done by Agrawal et al. [16]. This is probably due to a lack of knowledge about the health benefits of vitamin A in older children, on the part of their caregivers, or their perception of the ineffectiveness of VAS for older children, but these speculations have yet to be established by any qualitative study. A study conducted in Brazil reported a steady decline in the coverage rate of VAS program as the children grew older [19]. The literature suggests that poor VAS attendance is associated with low socio-economic status [20,21], which is in accordance with our study findings. One study in Bangladesh observed a close association between VAS coverage and household socio-economic status, determined by ownership of assets; reporting households without specific assets were more prone to miss VAS, indicating the presence of health inequity even for this important public health intervention [20]. Another paper from the Philippines suggests that those with a low household wealth index are less likely to receive VAS [20]. The National Micronutrient Status Survey of Bangladesh generated similar evidence, which shows that children from poorer wealth quintile families had less attendance to the VAS program over the preceding 6 months (70–76%) compared to those in higher quintiles (75–88%) [7]. These observations could also be due to the absence of knowledge, a lack of understanding of the importance of VAS or because of geographical barriers to seeking immunization services in the former group.

Bivariate analysis yielded significant differences in nutritional status between children who did and did not receive VAS in the last 6 months, although no such differences were observed in a multivariate analysis after adjusting for covariates. A higher proportion of children in the non-recipient group were wasted (WHZ <-2) or stunted (HAZ <-2) or underweight (WAZ <-2). Similar findings were reported in studies conducted in Bangladesh and Indonesia [21,22]. A systematic review suggested that VAD and severe acute malnutrition are strongly associated [23]. However, no significant differences were observed in the study conducted in Cambodia [17].

It is evident that families with unfavorable socio-demographic characteristics show poor attendance to most public health interventions. Our study also found that poor attendance to VAS was more common among children who did not receive the measles vaccine earlier in their life. During our study period, the national immunization program of Bangladesh concomitantly launched VAS and measles vaccinations [24], and this may be the reason for such observations of low coverage of VAS and measles vaccination. Similar findings were seen in another study done in the same setting [21]. Studies demonstrating that children missing VAS are also likely to miss immunization campaigns [22,25] indicate that some common socio-demographic characteristics might be responsible for poor compliance with these critical public health initiatives.

We did not find any difference in recipient status of VAS among male and female children, indicating a lack of gender inequity in VAS attendance. Similar findings have been reported from studies in India, Nepal and countries in Africa [14,15]. A study in Bangladesh and another one in the Philippines reported similar coverage patterns of VAS in male and female children [20,21].

It has been reported by several national dailies including the country’s most outspoken newspapers that vitamin A distribution is not reaching its maximum (more than 90% coverage) due to a lack of resource mobilization for distribution at the local level in sufficient quantities. In 2012 and 2013, despite mobilization of supplies by the Institute of Public Health and Nutrition, nationwide vitamin A distribution campaigns were disrupted following widespread rumors that many children fell sick after taking a high dose of vitamin A (200,000 IU). Due to such rumors, mothers were skeptical about participating in VAS campaigns with their children [26].

Another barrier to achieving uniform coverage of vitamin A was the absence of extensive promotion campaigns by field-level staff members working with health systems of Bangladesh due to geographical barriers, particularly in hard-to-reach areas. This was also a barrier to mothers in reaching the pre-set distribution posts, simply because of the lack of a transportation system or because available transports were too expensive for them to use, particularly at the time of harvesting and bad-weather days of the year. It was reported by Bangladesh demographic and health survey (DHS) that the level of VAS coverage varied across geographical regions of the country, and the lowest coverage was reported in Sylhet region where the majority of communities are located in hard-to-reach areas [10].

One clear finding from our observations is that the compliance of VAS is gradually increasing over time. Based on our results, the increase in VAS coverage may be due to a higher education level of mothers, their health care awareness, and better access to health care centers as well as improvements in health care delivery by the health system of Bangladesh.

The limitations of this study are the lack of a known denominator population as data were taken from a hospital-based surveillance system; also, information on vaccination status was based on mothers' recall that may have been affected by recall bias. The study did not take into account the distribution channel of VAS, which may not be efficient enough due to a lack of supplies or poor organization in service delivery, or reasons for lower compliance of the target population. However, we followed an unbiased systematic sampling of patients seeking diarrhoeal treatment irrespective of age, sex, socio-economic context, disease severity or nutritional status, and used a large data set for this analysis.

We believe our findings will encourage policymakers, public health personnel, and implementation researchers to explore the barriers to successful implementation of the VAS program, and will enable policymakers to address the shortcomings of the program to expand its coverage.

## Conclusion

Although the coverage of the vitamin A supplementation program in Bangladesh is improving, still one third of eligible children are lacking VAS. Efforts to reach this last mile may yield substantial benefits in reducing childhood mortality and morbidity. Our study revealed that non-compliance to VAS was associated with older children, uneducated parents, and low socio-economic status. To further increase coverage, special strategies are needed to target at-risk children who are currently deprived of supplementation.

## Paper context

This study aimed to determine the factors affecting low vitamin A coverage among the young children admitted to a diarrheal treatment facility. Results of the study indicate that low vitamin A coverage is associated with older children, parents without formal schooling, higher household poverty, low family income, and lack of measles vaccination. Thus, policymakers can formulate and implement specific programmatic approaches to prioritize at-risk children, which may enhance vitamin A coverage.
